# “Electroconvulsive Therapy: A Lifeline for Depression with Psychotic Features and Cognitive Decline” A case series

**DOI:** 10.1192/j.eurpsy.2025.1338

**Published:** 2025-08-26

**Authors:** M. Ligero Argudo, I. M. Peso Navarro, C. García Cerdán, C. Munaiz Cossío, C. Payo Rodríguez, R. K. González Bolaños, P. Andrés Olivera, E. Domínguez Álvarez

**Affiliations:** 1 Psychiatry, Clinical Hospital of Salamanca, Salamanca, Spain

## Abstract

**Introduction:**

Depressive disorders with psychotic symptoms in elderly individuals are serious conditions whose diagnosis may be complicated by confusion with neurocognitive disorders. Electroconvulsive therapy (ECT) is an effective intervention for these patients when pharmacological treatments are either ineffective or not feasible due to medical comorbidities.

**Objectives:**

To describe three clinical cases of women over 69 years of age with an initial diagnosis of depression with psychotic symptoms versus neurocognitive disorder.

To assess the clinical response to ECT during their hospitalization.

**Methods:**

A retrospective observational case series was conducted. Three female patients over 69 years old, admitted with a diagnosis of major depression with psychotic symptoms and signs of cognitive impairment, and who received ECT as part of their treatment, were included. The patients’ medical records were reviewed to gather information on their diagnosis, evolution, and response to treatment.

**Results:**

Case 1: Patient A (80 years old): psychomotor slowing, delayed response latency, nihilistic delusions with major affective symptoms. She received 10 sessions of ECT, with significant improvement in psychotic, depressive, and cognitive symptoms. She was discharged for outpatient follow-up.

Case 2: Patient B (70 years old): delusions of guilt, impersonation, and persecution, with concomitant major affective symptoms. She received 11 sessions of ECT, with significant improvement in affective, psychotic, and cognitive symptoms. Upon discharge, she continued follow-up with her Mental Health team.

Case 3: Patient C (72 years old): perplexed gaze, hypomimic facies, psychomotor slowing, thought blocking, no delusional symptoms, and major affective symptoms. She received 10 sessions of ECT, with little response in the affective and cognitive spheres. Care continued in the Convalescence Unit (subacute), and she was later institutionalized in a senior residence.

ECT was effective in two of the three patients in terms of psychotic, affective, and cognitive symptom response. In the third patient, where symptoms were more indicative of a neurocognitive disorder, ECT was ineffective, requiring long-term follow-up coordinated between Psychiatry and Neurology.

**Conclusions:**

ECT is effective in treating major depression with psychotic symptoms in elderly patients, although it may have limited response in cases of cognitive impairment. Therefore, a comprehensive approach and multidisciplinary follow-up are required to manage these cases.

**Disclosure of Interest:**

None Declared

